# Tumor-associated fibrosis: a unique mechanism promoting ovarian cancer metastasis and peritoneal dissemination

**DOI:** 10.1007/s10555-024-10169-8

**Published:** 2024-03-28

**Authors:** Hiroki Fujimoto, Masato Yoshihara, Raymond Rodgers, Shohei Iyoshi, Kazumasa Mogi, Emiri Miyamoto, Sae Hayakawa, Maia Hayashi, Satoshi Nomura, Kazuhisa Kitami, Kaname Uno, Mai Sugiyama, Yoshihiro Koya, Yoshihiko Yamakita, Akihiro Nawa, Atsushi Enomoto, Carmela Ricciardelli, Hiroaki Kajiyama

**Affiliations:** 1 Department of Obstetrics and Gynaecology, Nagoya University Graduate School of Medicine, Nagoya, Japan; 2 Discipline of Obstetrics and Gynaecology, Adelaide Medical School, Robinson Research Institute, University of Adelaide, Adelaide, Australia; 3 School of Biomedicine, Robinson Research Institute, The University of Adelaide, Adelaide, Australia; 4 Spemann Graduate School of Biology and Medicine, University of Freiburg, Freiburg im Breisgau, Germany; 5 Department of Obstetrics and Gynaecology, Kitasato University School of Medicine, Sagamihara, Japan; 6 Division of Clinical Genetics, Department of Laboratory Medicine, Lund University Graduate School of Medicine, Lund, Sweden; 7 Bell Research Center-Department of Obstetrics and Gynaecology Collaborative Research, Nagoya University Graduate School of Medicine, Nagoya, Japan; 8 Department of Pathology, Nagoya University Graduate School of Medicine, Nagoya, Japan

**Keywords:** Fibrosis, Extracellular matrix, Ovarian cancer, Metastasis, Mesothelial

## Abstract

Epithelial ovarian cancer (EOC) is often diagnosed in advanced stage with peritoneal dissemination. Recent studies indicate that aberrant accumulation of collagen fibers in tumor stroma has a variety of effects on tumor progression. We refer to remodeled fibrous stroma with altered expression of collagen molecules, increased stiffness, and highly oriented collagen fibers as tumor-associated fibrosis (TAF). TAF contributes to EOC cell invasion and metastasis in the intraperitoneal cavity. However, an understanding of molecular events involved is only just beginning to emerge. Further development in this field will lead to new strategies to treat EOC. In this review, we focus on the recent findings on how the TAF contributes to EOC malignancy. Furthermore, we will review the recent initiatives and future therapeutic strategies for targeting TAF in EOC.

## Introduction

Epithelial ovarian cancer (EOC) is the most lethal gynecologic malignancy [1]. Approximately 210,000 patients die annually from EOC globally, and the number is increasing every year [2]. Since ovaries are directly exposed to the intraperitoneal cavity, EOC frequently exhibits a characteristic mode of metastasis, such as peritoneal dissemination (metastasis to a wall of the abdominal cavity) and formation of omental cake (metastasis on the omentum) [3]. Due to the lack of specific symptoms and lack of an effective early detection screening system, more than half of the EOC patients are diagnosed in advanced stages with peritoneal dissemination [2]. Surprisingly, 2–7% of EOC patients in early stage (stages I–II) already have invisible micro-metastasis in the intraperitoneal cavity [4, 5]. Despite the use of combined cytoreductive surgery and platinum-based chemotherapy, over 70% of advanced-stage patients experience recurrence within 5 years [6], and its most common recurrence site of advanced EOC is the intraperitoneal cavity [7]. Once recurrence occurs, the patients have a poor prognosis due to chemotherapy resistance and it is difficult to remove all the tumors. Considering that the prognosis of EOC dramatically worsens from stage III [8], it may improve the refractory nature of advanced EOC if it becomes possible to control the intraperitoneal dissemination.

**Fig. 1:**
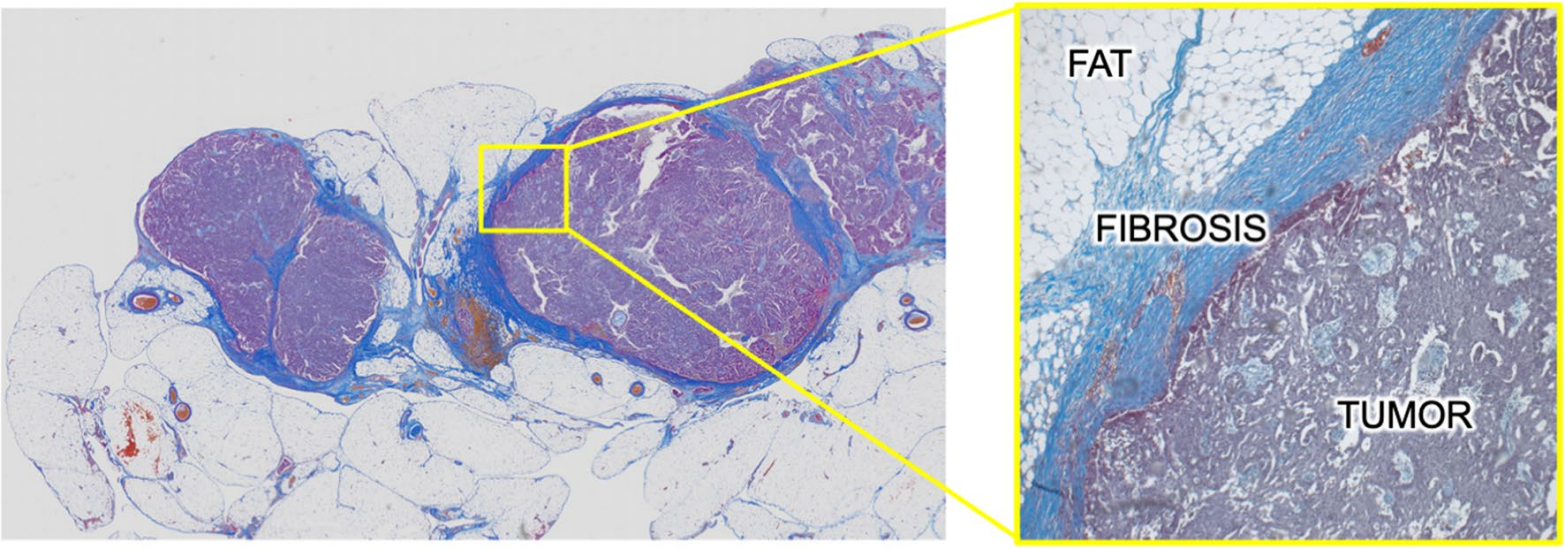
Example of Masson-trichrome staining in an omental metastasis from a patient with high-grade serous ovarian cancer (unpublished data). It shows complex structures of cancer cells with substantial fibrous stroma around the tumor

To control peritoneal metastasis, it is necessary to understand the underlying mechanism of intraperitoneal dissemination. The most common type of EOC, high-grade serous ovarian cancer (HGSOC) has been classified into four molecular subtypes based on mRNA profiling: immunoreactive, proliferative, differentiated, and mesenchymal [9, 10]. Among the four subtypes, the “mesenchymal” subtype, which involves dynamic stromal changes, has been reported to more frequently metastasize to the intraperitoneal cavity [11] and has the poorest patient prognosis [10, 12]. Therefore, understanding the nature and function of tumor stroma may help us to elucidate the mechanism of intraperitoneal dissemination of EOC. The tumor stroma is the main component of the tumor microenvironment which contains abundant extracellular matrix (ECM) molecules. Among ECM, collagen fibers are the predominant form of structural proteins, which play various roles in many types of solid tumor [13]. In fact, Masson-trichrome staining, which stains collagen fibers, shows omental metastases have complex structures of cancer cells with substantial fibrous stroma around the tumor cells (Fig. 1). We define this excessive accumulation of collagen fibers in tumors as tumor-associated fibrosis (TAF), which contributes to tumor progression, metastasis, and chemotherapy resistance of EOC so as with other neoplasms [13]. In general, fibrosis is seen in the wound healing process, which is precisely regulated by various cytokines and chemokines [14]. As cancer is described as “wounds that never heal” [15], both wound healing and cancer progression share common molecular reactions, which has been reviewed in many studies [16, 17], but how the intrinsic crosstalk with EOC cells and its surrounding fibrotic tissue contributes to tumor progression is still unclear. In this review, based on the recent findings, we focus on how TAF contributes to EOC’s malignancy. Furthermore, we review the recent initiatives and future therapeutic strategies for targeting TAF in EOC.

## Tumor-associated fibrosis: remodeled tumor-favorable stroma

Collagens are the most prominent molecules in ECM and considered one of the most influential factors in the nature of the stroma [13]. Since inappropriate accumulation and crosslinking of collagens have many downstream effects in many types of solid tumor, such as tumorigenesis, cell proliferation, invasion, metastasis, and dormancy, collagens have recently received significant attention [16, 18–21].

**Fig. 2:**
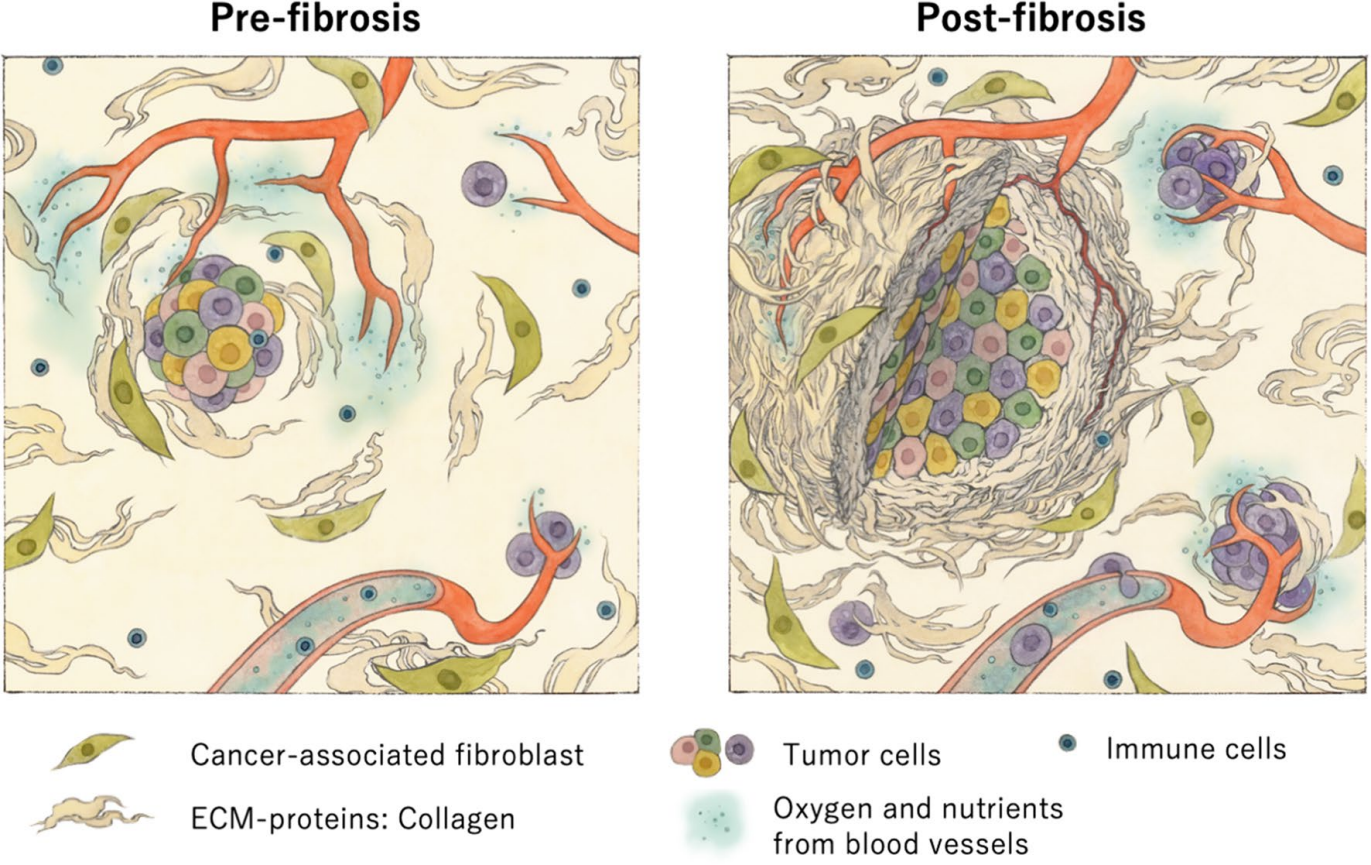
Schematic diagram of tumor-associated fibrosis. Left: pre-fibrosis. Right: post-fibrosis. Accumulation of collagen fibers alters tumor microenvironment by biochemical cross-talking with CAFs, which affects the nature of the tumors, such as tumor growth, angiogenesis, and immune cell infiltration. Abbreviation: ECM, extracellular matrix

Elucidating the mechanisms involved in remodeling the tumor surrounding microenvironment into TAF are key to controlling intraperitoneal dissemination (Fig. 2). Tumors opportunistically alter ECM homeostasis by biochemical cross-talking with cancer-associated fibroblasts (CAFs) and induce dynamic stromal change, thus affecting the nature of the tumors, such as tumor growth, angiogenesis, and immune cells infiltration [22, 23] (Fig. 2). ECM alteration in tumors consists of two major processes: degrading existing normal collagen fiber network in stroma, and creation of a tumor-microenvironment (TME) by cross-linking the collagen molecules [24].

Breaking down the normal collagen networks is catalyzed by matrix-degrading enzymes, such as ADAMs (a disintegrin and metalloproteinases), ADAMTSs (ADAMs with thrombospondin motifs), and matrix metalloproteases (MMPs) [25]. The MMP family are involved in various biological functions in tumors, such as tumor progression, and are considered to be a possible therapeutic target [25, 26]. In EOC, various types of MMPs are involved in tumor growth, invasion, and metastasis [27]. Interestingly, MMP-2 and MMP-9 may induce ascites formation via vascular endothelial growth factor (VEGF) secretion [28]. ECM also acts as a storage site for various cytokines, including VEGF and transforming growth factor-β (TGF-β), which are embedded in ECM and can be released by MMP proteolysis [29, 30]. These cytokines may be released to heal the damaged tissue when the wound or cancer invasion damage the stroma.

Lysyl oxidase (LOX) is a well-known collagen remodeling enzyme, which normally acts in the tissue repair by catalyzing the cross-linking of collagen fibers through oxidative deamination of lysine residues [31]. Accumulation of thick and long collagen fibers cross-linked by LOX reduces stromal elasticity and forms stiff tumor [32]. LOX and four LOX-like proteins (LOXL1-4) comprise the LOX-family, and recent studies indicate that elevated expression LOX family members is significantly correlated with tumor invasion, metastasis, and chemoresistance, which causes poor prognosis in EOC [33–35]. Furthermore, expression level of LOX1, LOX2, and LOX3 can predict prognosis and efficacy of platinum-based chemotherapy [36].

The elaborate and intricate collagen fiber network in tumors plays various roles in tumor malignancy. Recent studies have shown not only aberrant expression of ECM-remodeling enzymes but also the nature of remodeled stroma itself, such as stiffness and orientation of collagen fibers [37, 38]. In addition, tumor stroma contains various collagen molecules that form intricate collagen fiber networks, affecting various tumor functions, such as progression, invasion, and chemoresistance [13]. Next, we discuss the three distinct factors of ECM remodeling, which determines the stromal nature: the composition, the stiffness, and the alignment of collagen molecules.

## Composition of collagen molecules: various collagen molecules contribute to tumor malignancy

Collagen comprises 28 family members, each of which play unique functions in stromal architecture [39]. Surprisingly, recent studies revealed that various types of collagen molecules are associated with EOC progression and prognosis. Collagen type I is the most dominant collagen and mainly plays a structural role in the stroma. Accumulation and aberrant morphology of collagen type I in EOC stroma enhance tumor progression and metastasis [40]. Furthermore, its overexpression promotes EOC cell invasion with epithelial mesenchymal transition (EMT) and chemoresistance [41]. Collagen type I, α1 (COL1A1), is also considered a predictive marker for poor prognosis of EOC [42].

Collagen type II, α1 (COL2A), which normally augments strength in connective tissue, was also reported to be a predictive marker for recurrence in HGSOC [43]. It has been suggested that collagen type II secreted by stromal fibroblasts may promote tumor growth and angiogenesis [44].

Another type of collagen, collagen type IV(COL4), which is the main component of the basement membrane [45], also contributes to EOC cell function. Histopathological examination revealed that reduced expression of COL4 corelates with p53, Ki67, and EOC grade [46, 47], which indicates that EOC breaks down the basal membrane for further invasion. Tumor necrosis factor α (TNF-α) is one of the most significant promoter to cause basement membrane remodeling, paving the way for tumor invasion by decreasing COL4 [48]. Interestingly, high expression of COL4A2 is reported to lead to anoikis resistance via Notch3 signaling [49], by which EOC cells derived from epithelial cells can metastasize in the intraperitoneal cavity [50]. Furthermore, it has been suggested that adhesion and invasion may be promoted by collagen I and IV secreted by fibroblasts stimulated by TGF-β and other factors [45].

Recently, it has been demonstrated that high expression of collagen type VI, usually expressed in the basal membrane, directly affects tumor growth, invasion, and metastasis in various neoplasms [51]. In EOC, upregulation of collagen type VI α3 (COL6A3) may enhance tumor invasion and metastasis [52]. Furthermore, COL6A3 is also associated with cisplatin resistance in an autocrine manner [53]. Interestingly, a recent study indicated that chemotherapy induces the upregulation of various types of collagen, such as collagen type VI, in the omentum and peritoneum of EOC [54].

Minor types of fibrillar collagens are also reported to play several functions in EOC. Upregulation of collagen type XI α1 (COL11A1) appears to be related to platinum resistance via α1β1 integrin and discoidin domain receptor 2 (DDR2) [55]. Upregulation of collagens type XI by TGF-β enhances cell migration, invasion, and progression *in vivo* [56]. A recent report indicated that COL11A1 drives fatty acid β-oxidation, which may also facilitate cisplatin resistance [55]. Furthermore, Twist family primary helix-loop-helix transcription factor 1-related protein 1 (TWIST1) activated by COL11A1 induces chemoresistance and inhibits apoptosis in EOC cells [57]. Quantitative proteome analysis revealed the upregulation of COL12A1 in multidrug resistant EOC cell lines [58]. It is interesting that the expression of several types of collagen molecules in the ECM is either upregulated or downregulated in the chemo-resistant EOC cell lines. The biological function of collagen type XVIII is still not well-known, but expression of C-terminal fragment of this collagen, called endostatin, is significantly increased in EOC [59]. The function of collagen type XX is also poorly understood, but its expression is increased in various solid tumors, including EOC [59].

Aberrant accumulation of collagen fibers and expression of various types of collagens in the EOC stroma secreted by both cancer cell and fibroblasts can possibly affect the nature of the tumor. Interestingly, the expression pattern of collagen molecules, even the collagen type I, varies from report to report [40, 60]. This may possibly reflect the diversity of the stroma and suggests that expression of collagens is dynamically changing depending on the location and chronological stages of the EOC stroma.

## Stiffness: tough foundation for tumor invasion and metastasis

Recent studies suggest that biomechanical stress, such as stiffness, also drives various tumor functions [61]. In solid tumor, stiffness refers to resistance to deformation to an applied force, which is determined by the accumulation and crosslinking of ECM, such as hyaluronic acid and collagen type I [37, 62]. *In vitro*, the rigid substrate status increased proliferative capacity of EOC cells [63]. Virginie et al. reported that increased tumor stiffness with a high content of CAFs and collagen fibers promoted tumor growth *in vivo* in a mesenchymal HGSOC model [64]. Thus, the tumor stiffness also seems to influence the nature of EOC.

**Fig. 3:**
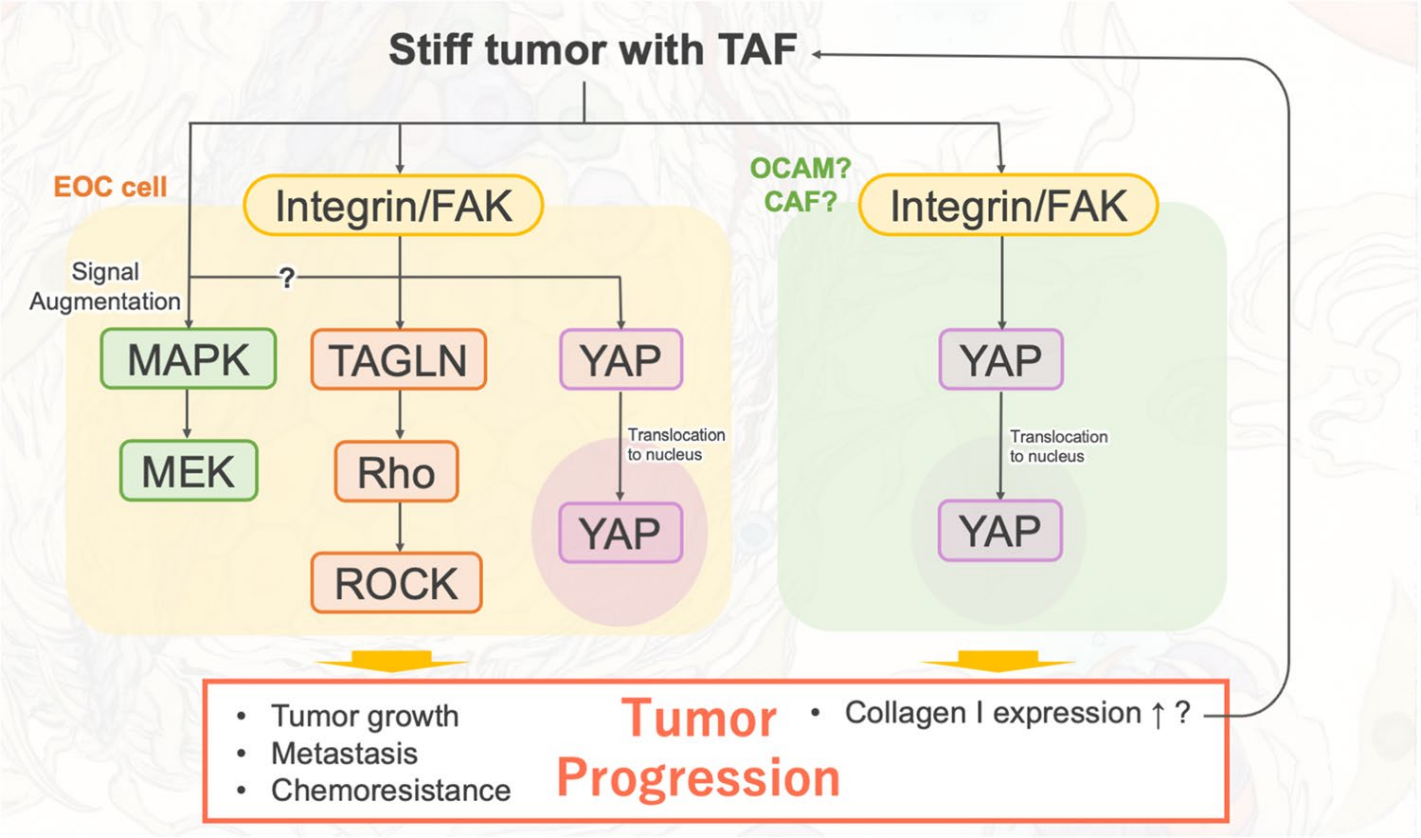
Tumor stiffness stimulates EOC cells by activating integrin/FAK and its subsequent signaling pathways: Rho/ROCK pathway, and Hippo pathway. MEK/MAPK pathway is also stimulated by stiff external stimuli, and it might be activated by integrin/FAK. These signal pathways responding to tumor stiffness facilitates tumor progression. Stiffness may also augment collagen production via Hippo pathway in OCAMs or CAFs. These reactions against tumor stiffness also promote tumor progression and more collagen production, augmenting tumor stiffness. Abbreviations: EOC, epithelial ovarian cancer; OCAM, ovarian-cancer associated mesothelial cell; CAF, cancer-associated fibroblast

As stiffness plays a vital role in EOC functions, several studies have been conducted to elucidate the detailed mechanism of how tumors receive and react to this biophysical signaling. The rigidity seems to stimulate multiple signaling pathways via integrins and FAK [54], and transmit external signals to various subsequent reporters (Fig. 3). The Yes-associated protein (YAP)/transcriptional coactivator with PDZ-binding motif (TAZ) is one of the well-known mechano-responsive signaling pathway regulators in various tumors, including EOC [37, 65]. Tumor stiffness promote nuclear translocation of YAP/TAZ in EOC cells and promotes proliferation, migration, EMT, and chemoresistance by receiving the external stimuli via focal adhesion kinase(FAK) [66–69], suggesting that rigidity influences various tumor functions via YAP/TAZ in the Hippo pathway. Interestingly, in a breast cancer model, the YAP related-pathway appears to form a positivistic feedback loop in CAFs to express additional collagen molecules forming more rigid tumors [70]. YAP promotes EMT and enhances the invasive and migratory potential of EOC cells [71]. Stiffness also activates RhoA/ROCK axis via transgelin (TAGLN), which is associated with larger cell morphology, augmented invasion, and cell proliferative capabilities [72]. In addition, a xenograft model of mesenchymal HGSOC showed increased activation of MAPK/MEK axis as tumor stiffness increased [64]. Together these studies indicate that the mechano-signaling pathway via integrin/FAK and its subsequent signaling pathways forms a very intricate signaling network in EOC, augmenting tumor malignancy (Fig. 3). The increased stiffness of the tumor may initially limit the ability of the cancer cells to escape from the tumor for further metastasis [73]. However, as tumors become stiffer, tumor cells acquire the capacity to facilitate their escape and metastasize [72]. Since stiff tumors can stimulate various signaling pathways related to cancer malignancy, targeting the one specific pathway might not be an effective way to control the tumor progression. However, softening tumor rigidity may restrict the various signal networks that respond to stiffness and limit the tumor progression.

Although it is not known whether same positive sequential cycle can be applied to intraperitoneal dissemination in advanced EOC, a comparison between the primary tumor and the metastatic tumor of HGSOC showed that the metastatic tumor was more rigid with higher expression of collagen type I and collagen-crosslinking enzyme, LOX [72]. Based on previous *in vitro* and *in vivo* reports, the response to stiffness may occur in both primary and metastatic tumors leading to enhanced tumor progression and activation of the signaling pathway shown in Fig. 3. Especially in the omentum, metastatic cancer cells may also interact with CAFs to augment collagen production [74], leading to increased stiffness and enhanced tumor growth. The mechanisms involved are discussed in more detail in the next section.

Research on signaling pathways responding to tumor stiffness in EOC is just beginning to emerge. A better understanding of mechano-signaling pathways their role in promoting stiffness of intraperitoneal metastatic tumors will lead us to find potential novel therapeutic targets in advanced EOC patients.

## Alignment of collagen fiber: metastatic rails for tumor invasion

The accumulation of fibrous collagens are major components of stiff tumors. The morphological changes in accumulating collagen fiber networks have recently attracted much attention and is a growing field of research [74]. Second harmonic generation imaging (SHG) has enabled us to visualize the collagen fiber morphology in the stroma. In 2006, the novel model of invasive breast cancer, tumor-associated collagen signatures (TACS) classification, was proposed based on the orientation of collagen fibers in tumor stroma observed by SHG [75]. There are many reports and reviews indicating that the process of remodeling a collagen network by degrading the wavy normal collagen fiber to forming straighter collagen fibers can pave the way for tumor invasion and intravasation [76–80]. Linear alignment of collagen fibers in the breast cancer is significantly associated with poor prognosis [81, 82], suggesting that orientation of collagen fibers may play a pivotal role in tumor function.

SHG examination also revealed aberrant collagen alignment in EOC [83, 84]. It has been reported that collagen fibers in the normal ovarian stroma exhibit a random mesh-like arrangement, whereas the alignment of collagen fibers in EOC becomes straighter [85]. In a study analyzing the arrangement of collagen fibers in the fallopian tube stroma, a possible origin of HGSOC, the collagen fibers network morphology was changed to a more a straight alignment as the disease progressed to malignancy, like in the TACS model of breast cancer [86]. The highly oriented collagen fibers promoted EOC cell migration more than the random collagen network in the normal stroma [87]. Although the precise mechanism how this alignment alteration affects the movement of cancer cells is not yet known, it may have an important influence on the nature of cancer. Interestingly, it has also been shown that the pattern of collagen fiber orientation differs depending on the histological subtype of EOC. Bruce et al. evaluated the collagen morphology in the stroma of three types of EOC: endometrioid, low-grade serous ovarian cancer (LGSOC), and HGSOC, using SHG, and it revealed that these three EOC subtypes have different collagen fiber morphology compared to normal ovary stroma [85]. Endometrioid EOC and HGSOC showed more straightforward collagen fiber networks, and LGSOC showed more fibrotic network compared to normal stroma with shorter fibers than HGSOC [85]. In breast cancer, deep learning analysis revealed that the percentage of straight collagens corelated with the stiffness of the tumor [88]. As discussed above, stiffness corelates with tumor aggressiveness and poor prognosis. This indicates that the content of straight collagen fibers in the metastatic tumor in intraperitoneal cavity can be a predictive marker for EOC patients. Although the mechanism and significance of collagen fiber linearization in EOC and its impact on tumor progression is still unclear, it is plausible that not only the composition and stiffness but also alignment of collagen fibers collectively affect multiple EOC functions in intraperitoneal metastasis. Furthermore, targeting collagen linearization can be a novel potential therapeutic approach for EOC. However, it is still unclear what causes collagen linearization in EOC. Interestingly, a recent report indicated that one of the WNT1 inducible signaling pathway (WISP) protein subfamily, WISP1, directly binds to collagen type I to induce linearization of collagen fibers *in vitro* and *in vivo* via the TGF-β axis in the breast cancer cell line, 4T1[89]. Increased expression of WISP1 was also observed in EOC patient tissue and correlates with poor prognosis of EOC patients [90], but the effects of WISP1 in the EOC microenvironment and its role especially fibrosis has not been fully investigated. As far as we know, no reports have focused on biophysical mechanisms that cause collagen linearization in EOC. Considering the collagen linearization plays a significant role in tumor progression, inhibiting the formation of linear collagen fibers should also be investigated as a novel therapeutic strategy for EOC.

## Omentum: the most common site for intraperitoneal metastasis of EOC

EOC is frequently associated with malignant ascites, in which spheroids spread throughout the intraperitoneal cavity and forms intraperitoneal dissemination. Omentum is the most common metastatic site in intraperitoneal cavity. For accurate staging of EOC and its therapeutic benefit, omentectomy is performed even in patients with early stage EOC [89]. The surgeons frequently observe dramatic changes in the omentum and desmoplastic reactions in advanced EOC [72], and it is assumed that the omentum play an significant role in EOC progression. The human omentum comprises layers of mesothelial cells and adipocytes with a highly vascularized structure. Collagen, elastin, and reticular fibers compose the central stroma with blood vessels and lymph vessels in this organ [91]. The omentum also has an unusual lymphatic circulation system. An early study showed that most of intraperitoneally administered dye, indigocarmine, reached and adhered to the omentum, which implies that omentum is the organ where intraperitoneally disseminated tumor cells are more likely to metastasize to [92]. As the omentum contains a lot of adipose cells, it is also considered as an energy storage site [91]. This organ is thought to be an energy provider for highly metabolic active cancer cells and a significant regulator of EOC metastatic growth [93].

**Fig. 4:**
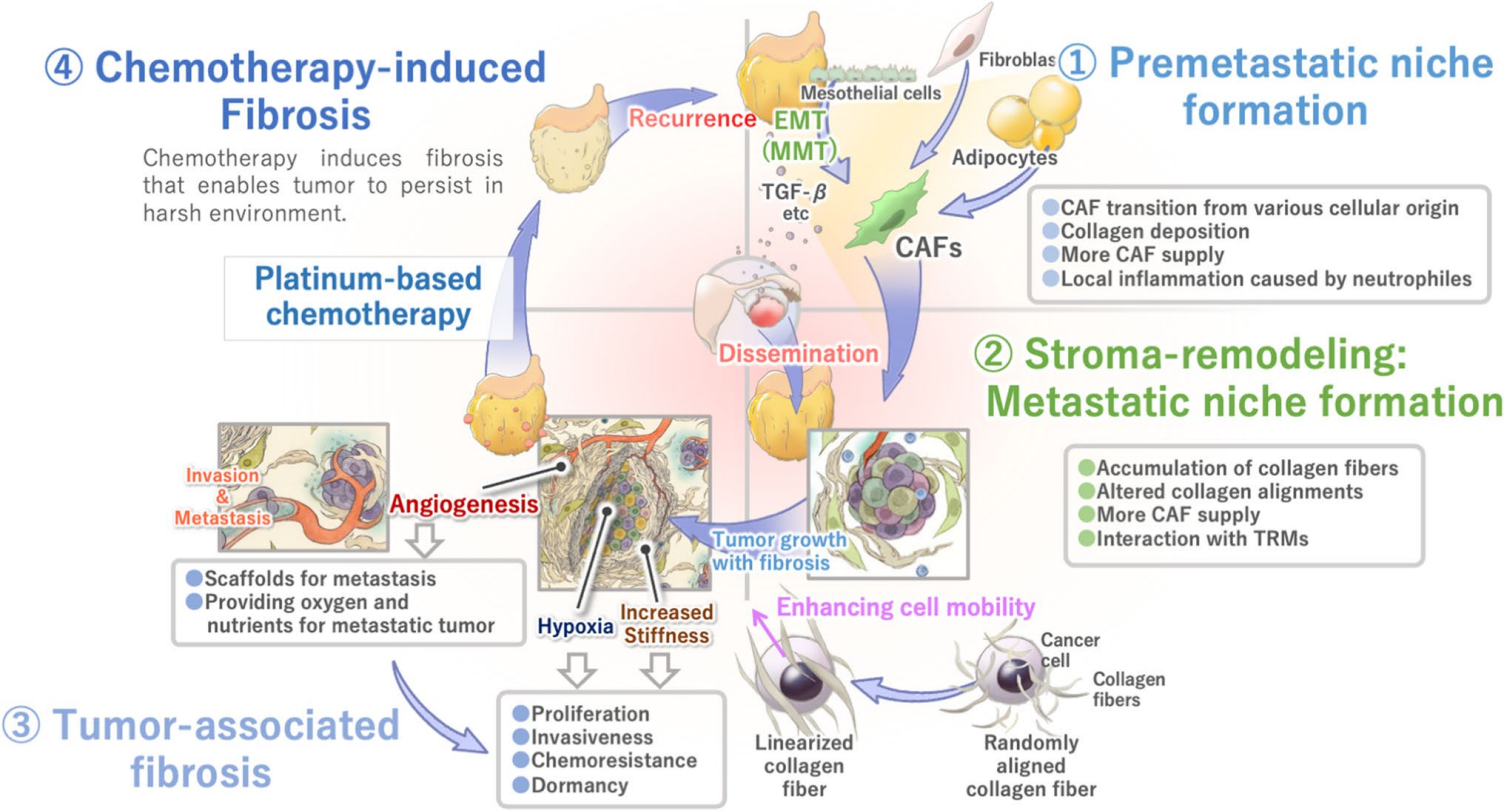
EOC metastasis and recurrence cycle of tumor stroma. 1 (top right): Chemokines and cytokines, such as TGF-β, and exosomes alter the stroma in future-metastatic sites. 2 (bottom right): After tumor cells attach to mesothelial cells, they establish the “soil” by remodeling the collagen-rich stroma. Expression patterns of various types of collagens, stiffness, and alignment of collagen are altered to create a tumor-favorable environment. 3 (bottom left): Tumor-associated fibrosis affects various tumor cell functions. 4 (top left): Chemotherapy induces further fibrosis, which may lead tumor cells into dormancy and enable them to persist in the harsh environment. After chemotherapy, tumor cells can sprout leading to recurrence. Abbreviations: EOC, epithelial ovarian cancer; EMT, epithelial mesenchymal transition; MMT, mesothelial-mesenchymal transition; CAF, cancer-associated fibroblast

The intraperitoneal cavity, including the omentum, also has a very characteristic local immune system [94]. Lymphoid tissue, called “milky spots” in the omentum, consists of lymphocytes, macrophages, and dendritic cells that contribute to peritoneal immunity [95]. Etzerodt et al. reported that tissue-resident macrophages (TRM) in omentum promoted intraperitoneal metastasis by forming a metastatic niche in EOC [96]. In pancreatic cancer, TRM also drives fibrosis, but whether the same reaction can be applied to the metastasis of EOC is still not clear. Immune response in tumors has attracted the interest of many researchers. Although we are not going in detail in this review, understanding of local immune response in the omentum might pave the way for elucidating the mechanism of creating a metastatic niche and intraperitoneal dissemination of EOC. Interestingly, the omentum is clinically considered a powerful source for regenerative surgery because of its biological functions [97]. These biological functions of omentum can provide a favorable environment for tumor progression. Figure 4 summarizes the co-evolution cycle of EOC cells and stroma in intraperitoneal metastasis and recurrence via omentum.

## Cultivating the fertile soil for future metastasis: forming a premetastatic niche in the omentum

As we discussed above, the altered collagen fiber composition, tumor stiffness, and collagen fiber arrangement in the tumor stroma can play a significant role in EOC progression. These changes of tumor microenvironment have been found to occur prior to formation of metastasis in various cancers, which is called pre-metastatic niche [98]. In EOC, a pre-metastatic niche refers to the formation of a microenvironment, in which EOC cells released from the primary tumor can easily attach and invade the peritoneum. Here, we will discuss the formation of premetastatic niches related to TAF in the omentum.

The first step of intraperitoneal metastasis is the attachment of EOC cells to the mesothelial cell layers in the abdominal cavity [99]. Although mesothelial cells function as a protective barrier against tumor attachment [45, 100], EOC is often associated with peritoneal dissemination [100]. Malignant ascites from the primary EOC flow through the abdominal cavity and can form a premetastatic niche scaffold for the attachment of the EOC cells [101]. One of the most significant cytokines in the malignant ascites is the TGF-β [102], which modifies the local microenvironment for tumor progression in various types of cancers [103–105]. In EOC, TGF-β stimulates various cells in the omentum to change into supportive cells for tumor progression (Fig. 4 top right). Mesothelial cells are one of the most abundant cells in the omentum and increase the expression of fibronectin, which promotes cancer cell adhesion by TGF-β stimulation [106, 107]. Furthermore, TGF-β causes EMT, which is also called mesothelial-mesenchymal transition (MMT), of the mesothelial cells, by which mesothelial cells express CAF markers, such as α-SMA, and also acquire mesenchymal functions [108, 109]. These changes in mesothelial cells may support the attachment and invasion of EOC cells on the peritoneum. We refer to these EOC-stimulated mesothelial cells that promote tumor progression as ovarian-cancer associated mesothelial cells (OCAMs). In addition to OCAM [108], TGF-β also promotes mesenchymal differentiation from fibroblasts [110] and adipocytes in the omentum [111, 112] to form CAFs. Because CAFs are already present in the omentum stroma even without metastasis in EOC patients [113] and 3D omentum model with CAFs increased the adhesion and invasion of EOC cells [110], CAF’s existence prior to the cancer cells’ attachment is important for metastasis. Since the omentum is mainly composed of adipocytes covered with abundant mesothelial cells, it can be a significant source of CAF, which may augment the tumors’ attachment and post-adhesive progression.

Recently, another cell communicative process between primary tumors and distant organs has been studied by many researchers to involve exosomes. Exosomes are small extravesicles (30–150 nm in diameter) secreted by various types of cells, and contain a variety of molecules, such as nucleic acids (mRNA, DNA, microRNAs), various proteins, and lipids, interacting with target cells [114]. Exosome released from primary EOC attach to the peritoneum, and promotes tumor progression [114]. Cell-to-cell communication via exosomes enhance immunosuppression, angiogenesis, CAF conversion, macrophage polarization, and mesothelial clearance, and thus creating a tumor-favorable microenvironment [115]. Although there are few reports that show the direct interaction between exosome and fibrosis of EOC, recent studies revealed that exosomes are strongly associated with fibrosis. Exosomes released from EOC cells are reported to promote the conversion of normal fibroblasts and adipose-derived mesenchymal stem cells to CAFs [116, 117], and CAFs with more activated mesenchymal signature [118]. Low contents of micro-RNA, miR-29c-3p, in exosome released from omental CAF may promote EOC metastasis by keeping continuous expression of MMP2 [119]. Since the exosomes contain various molecules and target multiple organs and cells [120], it seems that cancer cells and other non-cancer cells, such as CAFs, form more complicated networks than expected.

Immunity also plays a critical role in creating a tumor-associated microenvironment for metastasis. This is one of the growing fields in cancer research. Recent studies have shown that not only lymphocytes and macrophages but also neutrophils play an essential role in forming the premetastatic niche [121]. Ovarian tumor-derived inflammatory factors, such as interleukin-8, G-CSF, or MCP-1, stimulate neutrophils to secret neutrophil extracellular traps (NETs), which entraps tumor cells to attach to the omentum [121]. It also causes local inflammation, leading to fibrosis that creates an environment favorable for tumor invasion after attaching to the mesothelial layer of the omentum [121].

In this section, we showed how a primary tumor creates a supportive microenvironment for tumor attachment in distant organs through malignant ascites containing TGF-β and exosomes, which may support the subsequent growth of metastatic tumors. Next, we will discuss dynamic remodeling of tumor stroma and metastatic niche in the omentum from the perspective of TAF.

## Role of omentum as “soil” in metastatic tumor growth

Once EOC cells adhere to the mesothelial cells, they invade and grow in the omentum. For further cancer progression, the tumor cooperates with cells such as CAFs and TRMs to promote a tumor favorable microenvironment (Fig. 4 bottom right). Our previous histological analysis of the peritoneal metastasis diagnosed with advanced EOC revealed that fibroblastic cells surrounding the invading tumor cells were associated with the peritoneal mesothelial cells, CAFs, including OCAMs that cooperated together with cancer cells to form a microenvironment that promoted cancer cell invasion [122]. Furthermore, CAFs also secrete TGF-β, facilitating EMT of attached tumor cells, which helps the further invasion of EOC [123, 124]. CAFs are also be a major source of ECM materials, such as collagen, cytokines, and chemokines, for remodeling tumor stroma [54, 123]. Proteome analysis comparing the primary tumor and omental metastases revealed that increased expression of collagen type I and other ECM protein [54, 125]. Recently, it has been shown that the presence of COL11A-positive CAFs is associated with the presence of linear collagen fibers in EOC stroma [126]. COL11A expression is also increased in omental metastases compared to primary tumors [125]. Furthermore, the expression of WISP1, which linearizes collagen fibers in breast cancer, is significantly increased in advanced EOC patients [90]. Although there are no reports analyzing the collagen fiber arrangement in EOC metastases, the increased collagen observed in omental metastases [127] is predicted to be remodeled linearly like primary tumors to further support EOC cell invasion and growth.

As the metastatic tumor grows, it needs more blood supply because of the increasing demand of oxygen and other nutrients of the cancer cells [128]. Furthermore, excessive accumulation of collagen fibers makes the tumor more hypoxic and malnutrition by collapsing of the blood vessels due to the high interstitial pressure within the tumor [128] (Fig. 4 bottom left). Therefore, tumors change their metabolism by expressing glycolytic enzymes to tolerate malnutrition and a hypoxic environment known as the Warburg effect [128]. On the other hand, the tumor tries to break through this unfavorable hypoxic state via various biological pathways promoted by hypoxia-inducible transcription factor (HIF) axis [129]. The hypoxic microenvironment also affects mesothelial cells adjacent to tumor foci, causing collagen production through the HIF-α pathway and promoting further growth of intraperitoneal metastatic tumor [130].

Hypoxia created by tumor growth and TAF also induces VEGF expression, which controls oxygen and nutrients supply for tumor growth by promoting vascularization. It also provides a scaffold for invasion and metastasis [131]. In addition, Sonic Hedgehog (SHH) secreted from EOC cells promotes lymphangiogenesis via the Hh (Hedgehog)/VEGF-C signaling axis [131]. Since the cancer cells need guidance toward a new environment via blood vessels or lymph ducts, the tumor may remodel collagen fiber network, which promote metastasis through newly generated blood vessels [132]. Moreover, hypoxia also increases intracellular reactive oxygen species (ROS), which is typically generated during cell metabolism or inflammation, in the mitochondrial electron transport chain [133, 134]. ROS also play an important role in cancer progression in the tumor microenvironment [135]. Tissue analysis of EOC patients has shown the increased expression of NADPH oxidase 4 (NOX4), which produces ROS [135]. NOX4 is associated with TGF-β-mediated collagen production [4], suggesting that ROS may support collagen production in EOC through TGF-β. Furthermore, increased cellular ROS also promotes further EMT of EOC cells [35]. Thus, hypoxia contributes to the vicious cycle of tumor progression by mediating an intricated network of cytokines via ROS.

The omentum promotes metastatic tumor growth by the abundant CAFs and ECM supply, which is necessary for TAF formation. Interestingly, a recent analysis of the mesenchymal subtype of HGSOC reported that its expression signature profile was only present primary HGSOC in patients with concurrent upper abdominal/omental metastases but not in HGSOC that were confined to the ovary [136]. These findings suggest that the mesenchymal subtype may represent an advanced intraperitoneal tumor dissemination to the ovary rather than a subtype of primary HGSOC. EOC cells released from omental metastasis may have the capacity to change their overall tumor characteristics to a more malignant phenotype, depending on the microenvironment of surrounding cells and stroma at the metastatic niche.

In this section, we discussed the TAF creates a supplementary tumor microenvironment with increased rigidity, highly oriented collagen fibers, hypoxia, and vascularization for metastatic tumor growth after the EOC cells attached to the omentum. Next, we discuss the contribution of TAF to chemoresistance.

## TAF as biophysical barrier against chemotherapy and chemotherapy-induced fibrosis

Most EOC patients receive platinum-based chemotherapy as a first-line chemotherapy treatment after complete debulking surgery. Although this seems effective, over 70% of patients experience recurrence and develop platinum resistance within five years [137], contributing to the poor prognosis of EOC [138, 139]. So far, several mechanisms of chemoresistance have been identified in cancer cells, such as drug availability or signaling pathways [140]. Furthermore, EOC treatments targeting cancer cells have changed dramatically in recent years with the advent of molecularly targeted drugs, such as poly ADP-ribose polymerase (PARP) inhibitors, bevacizumab, and immunological checkpoint inhibitors, such as anti-programmed death 1 (PD-1)/programmed cell death-ligand 1 (PD-L1) [141], based on genetic profiling and platinum resistance [142]. However, the survival rate still has not improved dramatically [140].

Recently, it has been pointed out that the stroma plays a significant role in chemoresistance [143]. Fibrosis acts as a physical barrier to chemotherapy and provides an environment where tumors acquire chemotherapy resistance (Fig. 4 top left). As we discussed above, TAF creates an anaerobic environment within the tumor, which promotes HIF-1 expression leading to chemoresistance of tumor cells. HIF-1 also shifts tumor cells from apoptotic condition to dormancy, which also seems to promote cell survival under chemotherapy [144]. Furthermore, the dense deposition of collagen fibers also acts as a physical shield against chemotherapy by collapsing blood vessels, which reduces the amount of blood flow and chemotherapy drugs into the tumor [144–146].

Recently, Jeremy et al. proposed an intriguing model of platinum-resistant and sensitive recurrence taking into consideration with the function of ECM [147]. In the model, it is noted that the ECM protects cancer stem cells from chemotherapy by restricting the drug delivery into the tumor and maintains cancer cell heterogeneity [147]. If the platinum-sensitive tumor is covered with fibrotic stroma and protected from chemo drugs, it may be seemed like a platinum-resistant recurrence. Since the fibrotic microenvironment protects platinum-sensitive EOC cells from chemo drugs, platinum-based chemotherapy may be effective even for the patients who were diagnosed as platinum-resistant EOC if the tumor stroma becomes less fibrotic, which may be one of the reasons why platinum-based rechallenge therapy may be effective for some patients with platinum-resistant EOC [148]. Fibrosis also plays an essential role in tumor immune suppression by reducing infiltration of immune cells [149]. Recent studies have also shown that the effect of the anti-PD-1 treatment may be limited by the accumulation and cross-linking of collagen fibers by restricting T cell infiltration into the tumor [150].

Clinically, tissues exposed to chemotherapy are replaced by fibrotic stroma [150, 151]. This chemotherapy-induced fibrosis makes it challenging to complete cytoreductive surgery in advanced EOC [152] (Fig. 4 top left). However, there are few reports analyzing the fibrosis after chemotherapy. One histopathological studies of EOC after neoadjuvant chemotherapy (NAC) suggest that a high degree of fibrosis correlates with a better prognosis [153]. Although this report contradicts our viewpoint, but it only describes the degree of fibrosis after NAC, which may reflect the high efficacy of chemotherapy. An important factor is the composition of the environment surrounding the EOC cells that survive after chemotherapy as recurrence can occur if there are a few viable cells present. Factors associated with the fibrosis including stiffness, composition, and collagen linearity before and after NAC should be assessed in further studies. Chemotherapy-induced fibrosis can play various roles in EOC progression, but limited studies have investigated this so far.

We discussed above that TAF can act as a barrier against chemotherapy. In this context, reducing the fibrous stroma accumulation could be a breakthrough to enhance existing anti-tumor therapy and increase blood flow, chemotherapy-drug, and immune cell infiltration to the tumor. We propose that normalizing the fibrous stroma may provide a novel future therapeutic strategy. Lastly, we discuss the anti-fibrosis therapy on EOC.

## Is anti-fibrosis therapy a solution?

As discussed above, the tumor microenvironment plays a significant role in tumor proliferation, invasion, metastasis, and chemoresistance. Conversely, manipulating the microenvironment can affect various tumor functions. Interestingly, cancer cell proliferation ability was suppressed in the presence of normal stromal components [154]. So far, several stromal targets in EOC, such as endothelial cells, CAFs, and TAMs, have been identified [146]. Significantly, the microenvironment with an aberrant accumulation of collagen fibers influences various tumor functions, and TAF can determine a tumor’s fate. Conversely, reducing the fibrous tumor stroma may suppress tumor aggressiveness and improve drug delivery thereby improving therapeutic efficacy and patient prognosis. Several reports also show that anti-stromal therapy normalizes the malignant stroma and improves life expectancy in various cancer, such as pancreatic cancer, breast cancer, prostate cancer, hepatocarcinoma, and melanoma [155–160].

In terms of targeting fibrosis, there are two therapeutic ways: to suppress new fibrosis or to breakdown existing fibrosis. Since most EOCs are diagnosed at an advanced stage with intraperitoneal dissemination with fibrosis, reducing the fibrous stroma could effectively improve the prognosis of advanced EOC patients. If the TAF could be diminished, chemotherapy drugs could be delivered even into tumor foci where chemo drugs were not able to reach due to fibrous stroma. Several studies show several existing drugs which may control the TAF.

One of the target is the renin–angiotensin–aldosterone (RAA) system, which is associated with fibrosis in various organs and fibrotic diseases [161]. Primarily, angiotensin II promotes direct secretion of TGF-β and is considered to be a molecular driver of fibrosis [161]. Moreover, several studies have shown the effectiveness of the RAA system targeting therapies. For example, administration angiotensin inhibitor reduces solid stress due to fibrosis, improving the efficacy of chemotherapy in pancreatic cancer [128].

One of the candidates for anti-stromal therapy for EOC is angiotensin II type 1 receptors (AT1R) inhibitors that have been reported to inhibit tumor invasion, angiogenesis, and peritoneal dissemination [162]. Recently, adding the angiotensin inhibitor, losartan, to platinum-based chemotherapy reduced collagen fiber deposition and increased blood perfusion into the tumor, enhanced chemotherapy response, and reduced ascites formation [144]. Importantly, a retrospective analysis indicated that patients that received angiotensin system inhibitors had improved overall survival compared with patients who received other forms anti-hypertensives [144].

Metformin is another drug that reduces fibrosis of various organs, such as the heart, lungs, kidney, liver, and ovaries, via the TGF-β axis [163]. Intriguingly, an observational study of patients using metformin suggested that metformin reduced cancer risk by 23% [164]. After this report in 2005, increasing numbers of studies focused on how metformin decreases cancer risk and revealed its anti-cancer effects, including EOC [165, 166]. Furthermore, omental fibrosis is also strongly associated with insulin resistance, and anti-diabetic drugs such as metformin may play a role in reducing fibrosis [167]. Metformin affects various signaling pathways, mainly the AMPK pathway, which is involved in various tumor promoting properties [166]. Although metformin is considered to target cancer stem cells in its treatment [168], a recent study indicates that metformin suppresses EOC progression by inactivating stromal fibroblast stimulated by interleukin-6 (IL-6) [169], which cause fibrosis through chronic inflammation in acute peritoneal inflammation models [169]. Furthermore, a recent article indicated that metformin downregulates mesothelin (MSLN) expression of EOC cell lines, and downregulates IL-6/ STAT3 signaling activity that increases VEGF and TGF-β expression [170]. These reports indicate that metformin might inhibit tumor progression by mitigating stromal fibrosis. Recent phase II clinical trials indicate that the addition of metformin to treatments such as primary debulking surgery and chemotherapy in 38 patients with EOC improved overall survival and was associated with epigenetic changes in the tumor stroma [171].

In conclusion, understanding the complicated crosstalk between the tumor is necessary to break the vicious recurrence and metastasis cycle of EOC as mentioned in Fig. 4. The journey to develop the stromal-targeted therapies in EOC has just begun. In the future, further understanding of the TAF in EOC could lead to novel EOC treatment strategies (Table 1).

**Table 1:** Reported functions of collagens in epithelial ovarian cancer

Collagen	Biological function	Reported function in EOC	Reference
Collagen I	Fibrillar	Tumor growth, metastasis, EMT, chemoresistance, cell adhesion, invasion	[40, 41]
*COL1A*		Predictive marker for poor prognosis	[42]
Collagen II	Fibrillar	Tumor growth, angiogenesis	[44]
*COL2A*		Predictive marker for recurrence	[43]
*COL4A2*	Non-fibrillar	Anoikis resistance	[49]
*COL6A3*	Non-fibrillar	Tumor growth, invasion, Metastasis, Chemoresistance	[52, 53]
COL11A1	Fibrillar	Cell migration, invasion	[56]
*COL11A1*		Chemoresistance, anti-apoptosis	[55, 57]
*COL12A1*	Non-fibrillar	Chemoresistance	[58]
*COL18*	Non-fibrillar	Increased expression in tumor	[59]
Collagen XX	Non-fibrillar	Increased expression in tumor	[172]
